# Resting-State Functional Connectivity in Mathematical Expertise

**DOI:** 10.3390/brainsci11040430

**Published:** 2021-03-28

**Authors:** Miseon Shim, Han-Jeong Hwang, Ulrike Kuhl, Hyeon-Ae Jeon

**Affiliations:** 1 Department of Electronics and Information Engineering, Korea University, Sejong 30019, Korea; shim.miseon@gmail.com (M.S.); hwanghj@korea.ac.kr (H.-J.H.); 2 Interdisciplinary Graduate Program for Artificial Intelligence Smart Convergence Technology, Korea University, Sejong 30019, Korea; 3 Research Institute for Cognition and Robotics (CoR-Lab), Machine Learning Group Bielefeld University, 33615 Bielefeld, Germany; ukuhl@techfak.uni-bielefeld.de; 4 Department of Brain and Cognitive Sciences, Daegu Gyeongbuk Institute of Science and Technology (DGIST), Daegu 42988, Korea; 5 Partner Group of the Max Planck Institute for Human Cognitive and Brain Sciences at the Department for Brain and Cognitive Sciences, DGIST, Daegu 42988, Korea

**Keywords:** resting-state functional connectivity, mathematicians, expertise, neural efficiency, machine learning, support vector machine

## Abstract

To what extent are different levels of expertise reflected in the functional connectivity of the brain? We addressed this question by using resting-state functional magnetic resonance imaging (fMRI) in mathematicians versus non-mathematicians. To this end, we investigated how the two groups of participants differ in the correlation of their spontaneous blood oxygen level-dependent fluctuations across the whole brain regions during resting state. Moreover, by using the classification algorithm in machine learning, we investigated whether the resting-state fMRI networks between mathematicians and non-mathematicians were distinguished depending on features of functional connectivity. We showed diverging involvement of the frontal–thalamic–temporal connections for mathematicians and the medial–frontal areas to precuneus and the lateral orbital gyrus to thalamus connections for non-mathematicians. Moreover, mathematicians who had higher scores in mathematical knowledge showed a weaker connection strength between the left and right caudate nucleus, demonstrating the connections’ characteristics related to mathematical expertise. Separate functional networks between the two groups were validated with a maximum classification accuracy of 91.19% using the distinct resting-state fMRI-based functional connectivity features. We suggest the advantageous role of preconfigured resting-state functional connectivity, as well as the neural efficiency for experts’ successful performance.

## 1. Introduction

Recently, the differences between professional mathematicians and non-mathematicians have been investigated with respect to functional specificity and structural connectivity with functional magnetic resonance imaging (fMRI) and diffusion MRI [1,2]. Mathematicians’ high level of expertise in mathematics yielded a focal activation in the dorsolateral prefrontal cortex, whereas non-mathematicians recruited a broadly distributed brain network, including the left inferior frontal gyrus, frontal sulcus, intraparietal sulcus, and right inferior parietal lobule. Different levels of expertise were also reflected in the psychophysiological interaction, showing divergent connectivity among the precentral gyrus–putamen–caudate nucleus and the superior parietal lobule–precentral gyrus between the two groups. With respect to the architecture of white matter tracts, dorsal and cortico-thalamic structures were modulated by the level of mathematical expertise. The structural integrity of the arcuate fasciculus and superior longitudinal fasciculus was higher in mathematicians, whereas stronger cortico-thalamic connectivity was observed in non-mathematicians. These previous studies demonstrated expertise-dependent modulation on the functional specificity and anatomical connectivity using task-related fMRI and diffusion MRI, respectively.

Experts’ functional connectivity in large-scale brain networks, associated with high-level mathematical skill, is still called into question. Considering the pivotal roles of mathematical ability in learning and education [3], as well as in the prediction of academic achievement of children [4], a thorough investigation on the brain connectivity in association with mathematical expertise should not go unheeded. However, previous neuroimaging studies showed varying results because of their different task demands or diverse study designs, and this makes it difficult to compare those findings across studies. To avoid this problem, in the present study, we used resting-state fMRI, in which no specific tasks are needed.

Resting-state fMRI is known to play a pivotal role in understanding the functional connectivity of brain regions. It detects low-frequency blood oxygenation level-dependent (BOLD) signal fluctuations during rest, demonstrating connectivity between anatomically distinct but functionally related regions [5,6,7,8]. These spontaneous BOLD activities unveil intrinsic functional architectures such as visual networks, auditory networks, sensorimotor networks, default mode networks (DMNs), basal ganglia networks, language networks, executive control networks, and dorsal/ventral attention networks [7,9,10,11,12,13,14,15,16,17,18,19,20,21,22,23,24,25]. Interestingly, these intrinsic connectivity networks have been constantly observed within and across people [26].

Regions that activate together during specific functional tasks are known to be correlated among themselves during resting state as well, maintaining their traits of functional specificity. Many studies have suggested that spontaneous BOLD activities in resting state provide useful information on the human brain that is organized into multiple distinct yet inherently interactive regions that have specific functions [27]. Using this approach, researchers have elucidated how functional connectivity in resting state is related to large-scale brain networks that are allocated to specific cognitive functions [28]. Moreover, resting-state fMRI has been used to demonstrate group-specific features such as early adolescents’ network maturation [29], highly intelligent individuals’ functional connectivity and global efficiency [30,31], and individuals with better set-shifting functions showing positive resting-state connectivity between frontoparietal and visual networks [32]. Therefore, using resting-state fMRI enables us to understand the linked functions of anatomically separate areas through the correlation of resting BOLD activities with individual differences.

It should be noted that various factors such as types of methods (parametric or non-parametric), types of variables (discrete or continuous), or the sample size yield varying statistical results, even with identical fMRI data [33,34,35]. Therefore, the significance of the result, as well as its interpretation and generalizability to the population at large, should be examined carefully. With respect to this issue, previous studies suggested to use another type of analysis, that is, the machine learning approach, which can provide supporting information for the data interpretation and generalizability of fMRI-based results by classifying and predicting significant neuroimaging features [36,37,38]. For example, the difference between musical experts and non-experts was investigated using the machine learning approach, and the results showed a classification accuracy of 77%, implying that the significant features from fMRI data not only provide psychophysiological interpretation, but also grasp functional or structural differences between groups [38].

In the present study, we investigated how mathematicians and non-mathematicians differ in the correlation of their spontaneous BOLD fluctuations across brain regions during resting state, with the aim of delineating the functional network structures modulated by varying levels of mathematical expertise. To this end, we recruited two groups of participants, that is, mathematicians with a high-level of mathematical expertise and non-mathematicians as a control group, and we performed a group comparison using resting-state fMRI data. Most of the previous studies postulated specific regions of interests (ROIs), examined with which areas or networks the ROIs are correlated, and interpreted the results with respect to cognitive processes in which the ROIs are known to be involved. Therefore, interpreting these results is usually dependent on the functions of the predetermined ROIs. However, in the present study, we explored the resting-state functional connectivity in the whole brain network instead of selective ROIs, and thus we did not limit our investigation to subjectively selected areas. We hypothesized that the functional connectivity of mathematicians and non-mathematicians would be characterized by a set of brain regions reflecting the different levels of expertise in each group. In particular, mathematicians’ resting-state fMRI networks would be similar to task-based fMRI networks, which pertains to experts’ preconfigured connectivity and a neural efficiency. Finally, we expected that the functional networks would identify the mathematicians and non-mathematicians by the classification performances based on machine learning algorithms, which provides a vital piece of information about the different functional connectivity between the groups.

## 2. Materials and Methods

### 2.1. Participants

We analyzed the resting-state fMRI data of the participants, which were collected from a previous study in 2017 [1]. Two groups of participants were recruited—mathematicians and non-mathematicians, depending on their levels of expertise in mathematics. Participants in the mathematician group were first chosen on the basis of their occupations (i.e., mathematicians or mathematics teachers) from a pool of 53 adults and screened for mathematical competence via a standardized mathematics test (Mathematik-Test: Grundkenntnisse für Ausbildung und Beruf) [39]. Participants in the non-mathematician group were first recruited from a pool of 34 adults whose occupations were not related to the use of professional mathematics. They were also screened via the mathematics test, such that we could eliminate people whose level of mathematical expertise was similar to mathematicians’ level. In addition, we measured all of the participants’ general intelligence and verbal working memory using the Berlin Intelligence Structure Test [40] and the German version of the Wechsler subtest [41], respectively. Details are provided in Table 1, demonstrating that the two groups showed a significant difference only in the mathematical test. All of these behavioral data were obtained on separate days consecutively (Day 1 for the mathematics test, Day 2 for the general intelligence and verbal working memory tests, and Day 3 for resting-state fMRI scanning), such that we were able to minimize the participants’ fatigue and remove possible unwanted influence of the behavioral tests on resting-state fMRI data. All of the participants gave written, informed consent to participate in the study. The Research Ethics Committee of the University of Leipzig (approval number 953) approved the study in accordance with the Declaration of Helsinki.

### 2.2. Resting-State fMRI Acquisition

Resting-state fMRI data were acquired on a human whole-body 3 Tesla Siemens TIM TRIO (Siemens Healthcare, Erlangen, Germany) with a 32-channel head coil. 3D T1-weighted structural images were previously obtained (MP-RAGE sequence, non-selective inversion pulse, inversion time (TI) = 650 ms, repetition time (TR) = 1.3 s, time to echo (TE) = 3.93 ms, flip angle = 10°, bandwidth = 67 kHz/px, matrix = 256 × 240 m^2^, 128 sagittal slices, spatial resolution = 1 × 1 × 1.5 m^3^, two acquisitions). A T2*-weighted gradient-echo echo-planar imaging (EPI) sequence was used (TR = 2000 ms, TE = 30 ms, flip angle = 90°, field of view (FOV) = 64 × 64 m^2^, 30 slices, resolution: 3 × 3 × 4 m^3^, interslice gap = 0.8 mm, 420 volumes). The scanning time was 14 min, and participants were required to watch a fixation lying still in the scanner.

### 2.3. Resting-State fMRI Data Analysis

Resting-state fMRI data were preprocessed using FSL v5.0, Matlab R2017b and AFNI Version 17.2.17 (https://afni.nimh.nih.gov/ 27/03/2021). After removal of the first four volumes of each scan, the data were slice time-corrected. Head motion was quantified by frame-wise displacement (the sum of rotational and translational rigid body realignment parameters from one volume to the next) [42]. We first evaluated the number of volumes with a frame-wise displacement >0.5 mm for each participant individually. Then, the dataset that required the most elimination served as a standard for determining the number of volumes to be discarded for all the participants’ datasets, resulting in removing 37 volumes. Therefore, each participant had 379 volumes. Through this way, we were able to keep the amount of information added by each person identical, avoiding potential confounding effects from different degree of movement.

Subsequently, the individual T1-weighted magnetization-prepared rapid gradient-echo (MP-RAGE) images were used to generate partial volume maps for grey matter (GM), white matter (WM), and cerebrospinal fluid (CSF). To this end, the T1 data images were skull-stripped, aligned to Montreal Neurological Institute (MNI) standard space, and segmented using FSL’s fast [43]. Next, WM and CSF segmentations were thresholded at 80% tissue probability, and affinely aligned to individual space. To control for motion, as well as scanner-related and physiological noise, five principal components from WM and CSF were extracted from the functional data and regressed out together with the six linearly detrended motion parameters previously determined [44]. Finally, residual data were bandpass-filtered at 0.01–0.1 Hz, spatially smoothed with a 6 mm FWHM kernel, and affinely aligned to a T1-weighted MRI template derived from a normative adult population [45], as provided in SPM12 (Wellcome Imaging Department, University College, London, U.K., http://www.fil.ion.ucl.ac.uk/spm/).

Next, the resting-state fMRI time series samples were extracted using SPM12 (SPM12, http://www.fil.ion.ucl.ac.uk/spm/, Wellcome Trust Centre for Neuroimaging, London, U.K.) [46]. GM was segmented into 112 ROIs based on the Anatomical Automated Labeling (AAL) atlas [47] implemented in SPM12. The representative BOLD signals of each ROI were extracted using principal component analysis (PCA), where the first component only was used [48]. The functional connectivity between all possible pairs of ROIs was evaluated using Pearson correlation coefficient. A Fisher r-to-z transform was computed to normalize r values for group comparison (mathematicians vs. non-mathematicians) [49,50]. The difference of functional connectivity between the mathematician and non-mathematician groups was estimated using the independent permutation *t*-test (10,000 replicates, *p* < 0.01) to avoid type I error [51,52]. Moreover, we computed the correlation coefficients between functional connectivity and the mathematics scores in each group using Pearson’s correlation to investigate if there is a region-to-region connectivity depending on the knowledge of mathematics (i.e., mathematics scores).

In order to further investigate different neural substrates between the two groups from a machine learning point of view, we performed the classification using 46 pairs of normalized r values as feature candidates, which showed statistically significant differences between the groups from the independent permutation *t*-test. In spite of the statistical significance in these pairs of features, they do not always guarantee the best classification performance [53]. Therefore, we estimated classification performance by selecting most discriminative features, for which another independent *t*-test was applied to each of the 46 features. The feature vector showing the smallest *p*-value between the two groups was first used for differentiating the groups, and the feature vector that had the next smallest *p*-value was added to the first feature vector for classification. This procedure was repeated until all 46 feature vectors were tested for classification. The classification accuracy was independently evaluated for each feature set (1 to 46 features) using a support vector machine (SVM) classifier with leave-one-out cross-validation (LOOCV) to avoid the overfitting of the SVM classifier [53,54]. In the LOOCV, one sample (mathematician or non-mathematician) was used as the test the data, whereas the other samples were used as the training data, which was iterated until every sample was used as the test data. The raw brain data and analysis scripts will be made available on request.

## 3. Results

### 3.1. Functional Connectivity between Mathematicians and Non-Mathematicians

**Figure 1:**
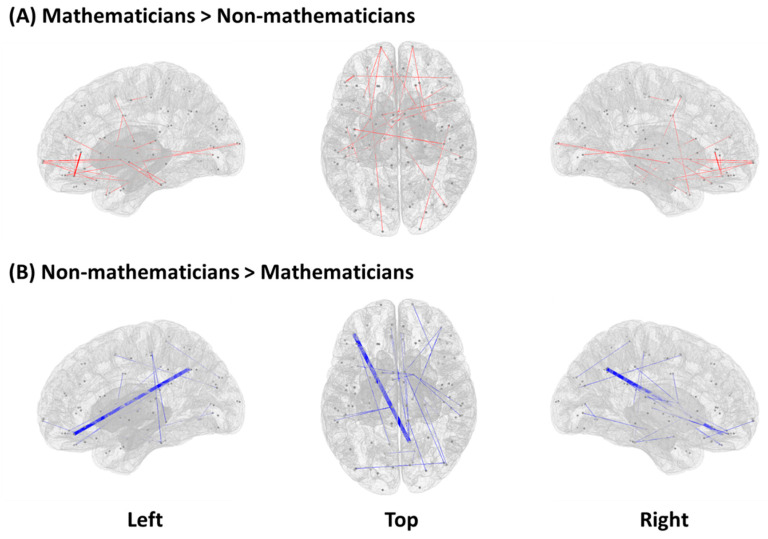
Significant differences between mathematicians and non-mathematicians in terms of functional connectivity (independent permutation *t*-test, *p* < 0.01). (**A**) Increased functional connectivity in mathematicians compared to non-mathematicians. (**B**) Increased functional connectivity in non-mathematicians compared to mathematicians. Line width is defined based on inverted *p*-values (1/*p*). Dots indicate 22 pairs of ROIs for mathematicians (A) and 24 pairs of ROIs for non-mathematicians (B). Left and right views of ROI pairs show sagittal images and top view demonstrates an axial image.

**Figure 2:**
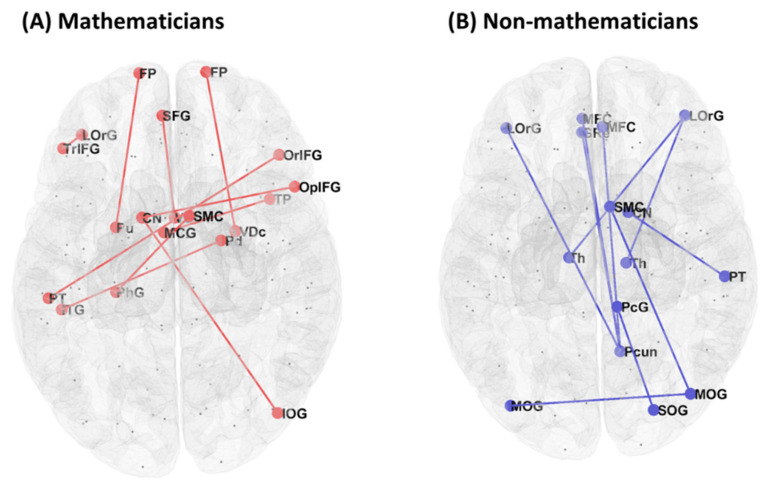
Connectivity of the top-ten ROI pairs selected from Table 2. ROI pairs are denoted with circles connected to one another for (**A**) the mathematician group and (**B**) the non-mathematician group.

Resting-state functional connectivity showed a significant group difference (mathematicians vs. non-mathematicians) in 46 pairs of ROIs (Table 2). The mathematician group, compared to the non-mathematician group, showed higher functional connectivity in 22 pairs of ROIs, while the non-mathematician group demonstrated higher functional connectivity in the other 24 pairs of ROIs. In Figure 1, we present a graphical illustration of all the functional connections in each group, where red and blue lines represent significantly higher functional connectivity in the mathematicians and non-mathematicians, respectively. Among these connections, we selected the top-ten connections and rendered them on the brain, demonstrating conspicuous differences in the connectivity patterns between the groups (Figure 2).

### 3.2. Linking Functional Connectivity to Mathematics Scores

**Figure 3:**
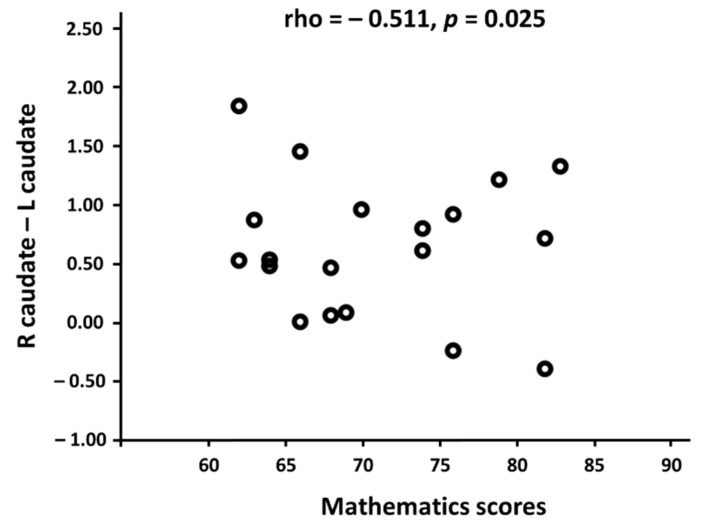
The relationship between the mathematics scores and the functional connectivity (normalized r values) between the bilateral caudate nucleus in the mathematicians. *X*-axis indicates the mathematicians’ scores in the standardized mathematics test and the *Y*-axis indicates the functional connectivity values between the left and right caudate nucleus using Pearson’s correlation coefficients. A significant negative correlation was found only in the mathematician group.

To investigate a crucial relationship between the brain’s functional network and mathematical competence, we performed Pearson’s correlation analysis between each of the 46 functional connectivities (Table 2) and the mathematics scores in mathematicians and non-mathematicians. As a result, the functional connectivity between the left and right caudate nucleus was negatively correlated with the mathematics scores in the mathematician group (r = −0.511, *p* = 0.025; Figure 3). In non-mathematicians, we could not find any significant results. This indicates that only mathematicians showed stronger functional connectivity between the left and right caudate nucleus as their mathematics scores became lower.

### 3.3. Classification Performance

**Figure 4:**
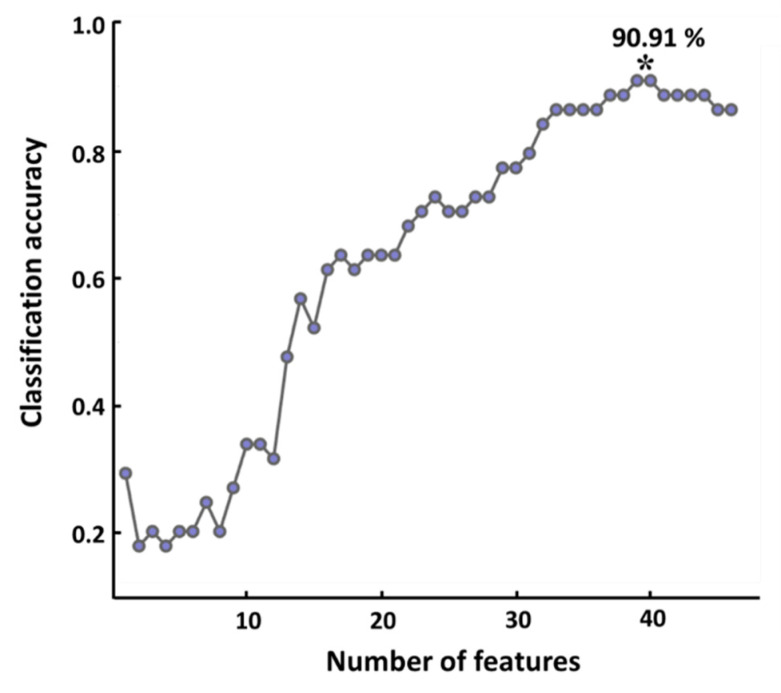
Classification accuracy with respect to the number of features. The maximum classification accuracy of 90.91% was obtained when using 39 features, which is denoted by an asterisk.

Figure 4 shows the changes in classification accuracy with respect to the number of features (pairs of ROIs). An increasing trend in classification accuracy was observed as the number of features used for classification increased, and the highest classification of 90.91% was obtained when most features (*n* = 39) were used.

## 4. Discussion

In the present study, the evidence of an expertise-dependent brain network was garnered from the disparate functional connectivity between mathematicians and non-mathematicians. The level of integration among brain regions at resting state, in other words, the resting-state functional connectivity, is deeply intertwined with neural architectures that support fundamental aspects of human behavior [14,55,56]. Much has been studied about changes to neural networks using resting-state fMRI and task-related fMRI, emphasizing which brain regions establish functionally specialized networks, how functional connectivity changes its strength, and how these changes link to alterations in cognitive functions, emotion, and behavior [26,57,58]. Indeed, researchers have suggested that intrinsic connectivity networks acquired from resting-state data are closely linked to individual characteristics such as IQ, personality, and cognitive functions [57]. In line with this, we demonstrated diverging involvements of the frontal–thalamic–temporal connections for mathematicians and the medial–frontal areas to precuneus and the lateral orbital gyrus to thalamus connections for non-mathematicians. Among mathematicians, those who had higher scores in mathematical knowledge showed a weaker connection strength between the left and right caudate nucleus, demonstrating the connections’ characteristics associated with mathematical expertise. Moreover, we achieved a maximum classification accuracy of 91.19% when differentiating mathematicians and non-mathematicians using the distinct resting-state functional connectivity features.

### 4.1. Resting-State Functional Connectivity for Detecting Group-Specific Features

Resting-state fMRI has been widely used to detect differences between specific participant groups or to depict group-specific features in their cognitive functions. For example, network maturation was observed in early adolescence not only in the DMN, but also in the central executive network, and with a significant positive correlation between intelligence quotient and the central executive network [29]. Adults with superior and average intelligence exhibited a significant group difference in the functional connectivity and global efficiency of the DMN [30]. Individuals with better set-shifting functions showed positive resting-state connectivity between frontoparietal and visual networks, whereas individuals with higher performance in general executive functions showed increased resting-state connectivity between sensory network and DMN [32]. Individuals’ ability in creative thinking was also reflected in the connectivity pattern of resting-state fMRI, showing that participants’ creativity scores were positively correlated with the strength of the network, including the DMN, as well as salience and executive brain networks [59]. In other words, resting-state fMRI unveils the related functions of anatomically distinct areas through the correlation of resting BOLD activities with behavior-specific traits, representing individuals’ differences in cognitive functions. In line with this, we conducted a detailed investigation into mathematical expertise using resting-state fMRI, making great strides toward the understanding of mathematicians’ functional architecture in the brain and the neural underpinnings of their exceptional performances.

### 4.2. Functional Connectivity in Mathematicians

The intrinsic connectivity of resting-state data has functional significance by encoding and supporting the consolidation of experiences, and thus, it even facilitates behavior performance [60]. This indicates that the connectivity observed from the resting-state data may implicate the possible roles of the involved networks. The mathematicians’ group revealed significantly enhanced resting-state connectivity between the frontal and subcortical regions compared to non-mathematicians. Among this constellation of brain areas, we found that the frontal and temporal areas were significantly linked to the caudate nucleus, putamen, and ventral diencephalon that have been known to be closely intertwined with expertise-dependent network [1,61,62].

Much evidence has described the role of the caudate nucleus as the formation of a stimulus–response association in the pursuit of moderating goal-directed behavior [63,64]. The stimulus–response association is one of the features that distinguishes experts from novices. It has been suggested that experts retrieve chunks, patterns, or templates that are made of stimulus–response associations stored in the long-term memory and apply them for their professional performance [65,66,67]. Therefore, the involvement of the caudate nucleus in our mathematician group may be pertinent to its supporting role of expert-specific operation with arithmetical knowledge structures stored in the long-term memory. The occipital gyrus that was connected to the caudate nucleus in the mathematician group is also construed as aiding expertise-related behaviors. Professional mathematicians are likely to retrieve arithmetical facts from stimulus–response associations instead of getting involved in actual calculation, and as a result, mathematicians compile mathematical chunks in the occipito-temporal visual cortex [68]. In line with this, the present study also showed the significant connection between the caudate nucleus and the right inferior occipital gyrus only in mathematicians.

The frontostriatal network, including the caudate nucleus, putamen, and thalamus (vental diencephalon), in mathematicians was another major finding in the present study. The caudate nucleus and putamen, together with the ventral precentral gyrus, were the key connections modulated by mathematical competence according to the functional connectivity measured by psychophysiological interactions (PPIs) [1]. In terms of the thalamus, the spontaneous thalamic activity obtained by resting-state functional connectivity supports the key role of the fronto-thalamic connections in expertise, showing that the connections between the thalamus and inferior/middle frontal gyrus were positively correlated with experts’ training time [69]. The thalamus, in association with the prefrontal cortex and caudate nucleus/putamen, have been adduced not only as a hub for integrating information across cortical networks [70], but also as a mediation of many cognitive functions, such as spatial visual processing, attention, memory, and decision making [71,72,73,74]. Therefore, the involvement of the thalamus in the form of fronto-thalamic connections among mathematicians may be indicative of its active involvement in mathematics, which resultingly expedites information processing within the network and facilitates mathematicians’ professional performance in mathematics.

Another noteworthy connection in mathematicians was observed in the medial temporal lobe linked to the basal ganglia and inferior frontal gyrus (pars orbitalis). The inferior temporal regions and inferior frontal gyrus were found to be sensitively activated to quantity- and mathematics-related concepts [75]. When professional mathematicians were involved in the processing of mathematical statements (e.g., algebra, analysis, topology, or geometry), they showed activation in the inferior temporal region, along with the bilateral intraparietal sulci and bilateral sites in the dorsolateral, superior, and mesial prefrontal cortex [76]. Therefore, we suggest that the resting-state functional connectivity in the medial temporal lobe demonstrates its supporting role of processing mathematics-relevant information that is substantiated by the active involvement of the temporal regions observed in task-evoked BOLD signals in previous studies.

### 4.3. Mathematicians’ Preconfigured Functional Connectivity for Their Expertise

How efficiently the functional connectivity of resting-state fMRI updates to that of task-related fMRI is known to be closely associated with individuals’ level of performance, suggesting that the brain’s functional networks at rest need to be reconfigured when they are given specific tasks, getting ready for the successful performance of the tasks [77]. The fast and efficient reconfiguration and update of the functional network from rest to task is strongly interwoven with individuals’ task performances. Moreover, the degree of similarity between resting-state functional connectivity and task-related functional connectivity exerts on task performance [77,78]. For example, in high-performing individuals, their functional connectivity of resting state did not change much compared to that of task-related fMRI [77]. This reconfiguration efficiency or preconfigured functional connectivity during resting state, specifically with the similarity to a task-related configuration, has been known to aid in updates in task-related networks and predictions of individuals’ better task performance. Conforming to this, we also found that the resting-state functional connectivity in mathematicians was similar to the task-related functional connectivity when the same group of mathematicians had been involved in the processing of arithmetic calculation [1]. Mathematicians’ task-based functional patterns were observed in the areas composed of the inferior, middle, and superior frontal gyrus and the inferior occipital gyrus, which were also found in the resting-state networks in the present study. On the contrary, non-mathematicians showed substantially different functional networks between resting-state and task-related fMRI. Their functional activations were observed mostly in the inferior parietal lobule, bilateral occipital gyrus, superior frontal gyrus, and superior medial gyrus [1], whereas the resting-state functional connectivity in the present study was composed of the connections largely in the medial frontal gyrus to precuneus and lateral orbital gyrus to thalamus. Therefore, our findings reconcile with the argument that experts’ preconfigured resting-state functional connectivity, which is already similar to task-related functional connectivity even at rest, enables individuals to update their mental states for task performance more efficiently, leading to better behavioral performances compared to non-experts.

### 4.4. Neural Efficiency Correlated with the Functional Connectivity in the Caudate Nucleus

Neural efficiency, that is, “brighter individuals use their brains more efficiently when engaged in the performance of cognitively demanding tasks than less intelligent people do” [79], has been represented as having less brain activation and a shorter-range connectivity. Experts have been known to conserve mental resources that may have usually been served in non-experts for compensating their lack of proficiency, resulting in less neural activity in experts compared with non-experts. In the present study, mathematicians showed the negative correlation between mathematics scores and the strength of functional connectivity in the bilateral caudate nuclei (Figure 3), which indicates that the higher scores mathematicians achieved, the weaker functional connectivity they showed between the left and right caudate nucleus. This finding was also substantiated in several neuroimaging studies investigating the roles of the caudate nucleus in terms of the level of task performance or expertise. For example, the activity in the caudate nucleus increases when one is involved in effortful and complex tasks, whereas the caudate nucleus responds less in relatively easy and non-effortful tasks [80]. Pilots with a high level of expertise, compared to those with a moderate level of expertise, demonstrated less activity in the bilateral caudate nucleus with better performance in a simulated landing-decision task [62]. To summarize, the weaker functional connectivity is adduced to explaining the higher levels of expertise among our mathematicians, demonstrating how the connectivity in caudate nucleus changes with respect to ones’ expertise.

### 4.5. Classification Accuracy

Statistical analyses of functional or structural group differences have been widely used to investigate fMRI-based neuroimaging features, thereby revealing psychophysiological traits of specific population groups [1,2,81]. In the present study, together with this approach, we employed the machine learning technique to discriminate between professional mathematicians and non-mathematicians in their functional connectivity, using significant features extracted from resting-state fMRI data. We obtained a high classification accuracy of 91.19%, thereby cross-validating our statistical results from the aspect of machine learning. Particularly, we suggest that using resting-state functional connectivity features would play an important role in obtaining a relatively high classification accuracy. Indeed, several previous studies reported that functional connectivity features, compared to BOLD activities, showed better classification performances [82,83,84]. Therefore, it is necessary to look into functional connectivity from the resting-state fMRI data, as well as the various patterns of BOLD activations to understand the brain’s complex processes [85,86,87].

The machine learning approach can be used to develop practical fMRI-based applications. For example, as mathematical ability is closely linked to learning ability [3], discriminating between different levels of mathematical proficiency with a high classification accuracy may be an appropriate way to evaluate individuals’ learning capability and to provide educational feedback depending on their performances over the course of learning. To develop such an educational application, it would be better to use a large sample size in each group in the future study.

### 4.6. Limitations

There are a few caveats in our study. One could bring about the issue of several measurements of resting-state fMRI instead of a single measurement (as we did in the present study). Unless there are specific aims (e.g., comparing changes in resting state over several days or over various sessions before/after a certain experimental condition), researchers normally measure a single session for resting-state fMRI. However, it would also be possible to obtain several sessions of resting-state data and compare their results with the data from a single session, to see if there are any interesting differences between them for a future study. Another limitation was that, even though the mathematics test showed statistical significance between the two groups (mathematicians vs. non-mathematicians), some of the demographic factors such as age, years of education, and intelligence test (Table 1) showed a non-significant but still marginal difference, which might have influenced our results. Therefore, this should be considered more carefully in future studies to reveal group differences more precisely.

## 5. Conclusions

We elucidated how varying levels of expertise were reflected in the functional connectivity among various brain regions, by comparing mathematicians and non-mathematicians using resting-state fMRI. Moreover, with the help of the classification algorithm in machine learning, we also found that the resting-state fMRI networks between the two groups were different depending on features of functional connectivity. Our results showed diverging involvement of the expertise-specific functional connections in mathematicians, suggesting the advantageous role of preconfigured resting-state functional connectivity, as well as the neural efficiency for experts’ successful performance.

## Figures and Tables

**Table 1:** Demographic and cognitive profiles of the mathematicians and non-mathematicians.

	Mathematicians	Non-Mathematicians	Statistics
Age	33.42 (5.62)	27.23 (8.21)	*p* = 0.081
Gender, M/F	16/5	14/9	*p* = 0.276
Handedness, LQ	92.45 (3.65)	90.28 (8.25)	*p* = 0.269
Years of education	19.5 (2.7)	16.21 (6.28)	*p* = 0.079
Mathematics test	70.95 (7.13)	40.71 (7.69)	*p* < 0.001
Intelligence test	115.91 (12.35)	124.27 (15.23)	*p* = 0.072
WM (forward)	8.9 (3.12)	9.12 (4.2)	*p* = 0.319
WM (backward)	7.3 (1.9)	7.62 (1.59)	*p* = 0.273

Values depict mean (and standard deviation); statistics were obtained from independent *t*-tests, except for gender (Pearson’s chi-square test). LQ, laterality quotient); WM, working memory.

**Table 2:** List of region of interest (ROI) pairs showing statistically significant differences between mathematicians and non-mathematicians in terms of functional connectivity.

1st ROI	2nd ROI	*p*-Value
**Mathematicians > Non-mathematicians**		
Left lateral orbital gyrus (LOrG)	Left triangular part of the inferior frontal gyrus (TrIFG)	0.0003
Right ventral diencephalon (VDc)	Right frontal pole (FP)	0.0011
Left ventral diencephalon (VDc)	Left superior frontal gyrus (SFG)	0.0019
Left caudate nucleus (CN)	Right opercular part of the inferior frontal gyrus (OpIFG)	0.0021
Left parahippocampal gyrus (PhG)	Right supplementary motor cortex (SMC)	0.0031
Left caudate nucleus (CN)	Right inferior occipital gyrus (IOG)	0.0039
Right pallidum (Pd)	Left inferior temporal gyrus (ITG)	0.0039
Left middle cingulate gyrus (MCG)	Right temporal pole (TP)	0.0039
Left putamen (Pu)	Left frontal pole (FP)	0.0041
Right orbital part of the inferior frontal gyrus (OrIFG)	Left planum temporale (PT)	0.0041
Left putamen	Left inferior temporal gyrus	0.0051
Left ventral diencephalon	Right frontal pole	0.0051
Right postcentral gyrus	Left precentral gyrus	0.0059
Left fusiform gyrus	Left planum temporale	0.0061
Left anterior orbital gyrus	Left occipital pole	0.0061
Right putamen	Left frontal pole	0.0063
Left lateral orbital gyrus	Right triangular part of the inferior frontal gyrus	0.0065
Left frontal pole	Left posterior orbital gyrus	0.0065
Right occipital pole	Right planum temporale	0.0069
Right putamen	Right medial orbital gyrus	0.0073
Left amygdala	Right medial frontal cortex	0.0079
Right middle temporal gyrus	Left temporal pole	0.0081
**Non-Mathematicians > Mathematicians**		
Left lateral orbital gyrus (LOrG)	Right precuneus (Pcun)	0.000
Right thalamus (Th)	Right lateral orbital gyrus (LOrG)	0.0007
Left thalamus (Th)	Right lateral orbital gyrus (LOrG)	0.0011
Right middle occipital gyrus (MOG)	Right supplementary motor cortex (SMC)	0.0017
Right middle occipital gyrus (MOG)	Left middle occipital gyrus (MOG)	0.0021
Left gyrus rectus (GRe)	Right precuneus (Pcun)	0.0027
Right postcentral gyrus (PcG)	Right superior occipital gyrus (SOG)	0.0031
Right medial frontal cortex (MFC)	Right precuneus (Pcun)	0.0033
Right caudate nucleus (CN)	Right planum temporale (PT)	0.0037
Left medial frontal cortex (MFC)	Right precuneus (Pcun)	0.0041
Right ventral diencephalon	Right fusiform gyrus	0.0050
Right caudate nucleus	Left middle cingulate gyrus	0.0053
Left lateral orbital gyrus	Left precuneus	0.0061
Right caudate nucleus	Left anterior insula	0.0067
Right lingual gyrus	Left lingual gyrus	0.0067
Left precentral gyrus	Left middle temporal gyrus	0.0069
Left inferior temporal gyrus	Left precentral gyrus	0.0069
Left ventral diencephalon	Right fusiform gyrus	0.0075
Right gyrus rectus	Right precuneus	0.0081
Right hippocampus	Right inferior occipital gyrus	0.0089
Right caudate nucleus	Right supramarginal gyrus	0.0091
Right caudate nucleus	Left caudate nucleus	0.0093
Right calcarine cortex	Right lingual gyrus	0.0099
Right frontal pole	Right posterior orbital gyrus	0.0099

## Data Availability

The datasets used and analyzed during this study are available from the corresponding author on reasonable request.

## Abbreviations

CN—caudate nucleus; FP—frontal pole; GRe—gyrus rectus; IOG—inferior occipital gyrus; ITG—inferior temporal gyrus; LOrG—lateral orbital gyrus; MCG—middle cingulate gyrus; MFC—medial frontal cortex; MOG—middle occipital gyrus; OpIFG—opercula part of the inferior frontal gyrus; OrIFG—orbital part of the inferior frontal gyrus; Pcun—precuneus; PcG—postcentral gyrus; Pd—pallidum; PhG—para hippocampal gyrus; Pt—putamen; PT—planum temporale; Th—thalamus; TP—temporal pole; TrIFG—triangular part of the inferior frontal gyrus; SFG—superior frontal gyrus; SMC—supplementary motor cortex; SOG—superior occipital gyrus; VDc—ventral diencephalon.
